# Trigeminal nociceptive function and oral somatosensory functional and structural assessment in patients with diabetic peripheral neuropathy

**DOI:** 10.1038/s41598-018-37041-4

**Published:** 2019-01-17

**Authors:** Y. M. Costa, P. Karlsson, L. R. Bonjardim, P. C. R. Conti, H. Tankisi, T. S. Jensen, J. R. Nyengaard, P. Svensson, L. Baad-Hansen

**Affiliations:** 1 Department of Physiological Sciences, Piracicaba Dental School, University of Campinas, Piracicaba, Brazil; 2 Scandinavian Center for Orofacial Neurosciences (SCON), Aarhus, Denmark; 3 Danish Pain Research Center, Department of Clinical Medicine, Aarhus University, Aarhus, Denmark; 4 Core Center for Molecular Morphology, Section for Stereology and Microscopy, Department of Clinical Medicine, Aarhus University, Aarhus, Denmark; 5 Department of Biological Sciences, Bauru School of Dentistry, University of São Paulo, Bauru, Brazil; 6 Department of Prosthodontics, Bauru School of Dentistry, University of São Paulo, Bauru, Brazil; 7 Department of Clinical Neurophysiology, Aarhus University Hospital, Aarhus, Denmark; 8 Centre for Stochastic Geometry and Advanced Bioimaging, Aarhus University, Aarhus, Denmark; 9 Section of Orofacial Pain and Jaw Function, Department of Dentistry and Oral Health, Aarhus University, Aarhus, Denmark; 10 Department of Dental Medicine, Karolinska Institutet, Huddinge, Sweden

## Abstract

This case-control study primarily compared the trigeminal nociceptive function, the intraoral somatosensory profile and possible structural nerve changes between diabetic peripheral neuropathy (DPN, n = 12) patients and healthy participants (n = 12). The nociceptive blink reflex (nBR) was recorded applying an electrical stimulation over the entry zone of the right supraorbital (V1R), infraorbital (V2R) and mental (V3R) and left infraorbital (V2L) nerves. The outcomes were: individual electrical sensory (*I*_0_) and pain thresholds (*I*_P_); root mean square (RMS), area-under-the-curve (AUC) and onset latencies of R2 component of the nBR. Furthermore, a standardized full battery of quantitative sensory testing (QST) and intraepidermal nerve fibre density (IENFD) or  nerve fibre length density (NFLD) assessment were performed, respectively, on the distal leg and oral mucosa. As expected, all patients had altered somatosensory sensitivity and lower IENFD in the lower limb. DPN patients presented higher *I*_0_, *I*_P_, RMS and AUC values (p < 0.050), lower warm detection thresholds (WDT) (p = 0.004), higher occurrence of paradoxical heat sensation (PHS) (p = 0.040), and a lower intraoral NFLD (p = 0.048) than the healthy participants. In addition, the presence of any abnormal intraoral somatosensory finding was more frequent in the DPN patients when compared to the reference group (p = 0.013). Early signs of trigeminal nociceptive facilitation, intraoral somatosensory abnormalities and loss of intraoral neuronal tissue can be detected in DPN patients.

## Introduction

Diabetic peripheral neuropathy (DPN) is a well-known complication and is estimated to occur in 10–90% of type 1 and/or type 2 diabetes patients^1^. However, in contrast to manifestations in the distal parts of especially the lower extremities, potential orofacial neurophysiological consequences are not adequately explored. The previous lack of focus on the orofacial complications to diabetes may possibly be explained by professional demarcations between dentistry and medicine^2^. However, adequate trigeminal sensory function is of crucial importance for the quality of life and well being of patients, e.g., efficient mastication, enjoyment of food, communication and intimacy.

Since the beginning of the 21^st^ century, techniques and methods for comprehensive investigation of the trigeminal system with focus on sensory function in general, and nociceptive function in particular, have become more accessible and accepted in the clinical and research environment^3–5^. This is mainly due to extensive work on establishing the validity and reliability of techniques for the orofacial region assessment, e.g. nociceptive blink reflex (nBR)^6–9^ and quantitative sensory testing (QST)^10–13^. The nBR is an electrophysiological test that can be used to evaluate the trigeminal nociceptive function with the aid of concentric surface electrodes that yield a more selective activation of nociceptive fibres^9^, whereas QST encompasses a standard battery of psychophysical tests that provide a comprehensive phenotyping of the somatosensory function^12^. Some advantages of these neurophysiological and psychophysical tests are the precise quantification of the neuronal function and their potential to detect early stages of sensory dysfunction^14^.

It is also important to emphasize that they do not replace bedside clinical examination, but rather provide additional information that may help in the diagnosis and treatment choice^3,15^. Notably, there is solid evidence in favour of the clinical value of nBR and intraoral QST assessment^5,7,16^. In addition, structural analysis of the tissues, e.g., intraepidermal nerve fibre density (IENFD) and nerve fibre length density (NFLD), can also be considered of great value in the diagnosis of small fibre damage in neuropathic pain patients^17^. Although previous evidence has already found indications of abnormal masseter inhibitory and “jaw jerk” reflexes^18^ and prolonged R1 and contralateral R2 responses of blink reflex^19,20^ in patients with DPN, which could indicate subclinical cranial neuropathy, no systematic combined investigation of possible orofacial neurophysiological, somatosensory and structural consequences of DPN has been undertaken so far.

Based on the above, the primary aims of this study were to compare the trigeminal nociceptive function, the intraoral somatosensory profile and structural nerve changes between DPN patients and healthy participants. In addition, we also correlated the intraoral QST with (a) the QST of the lower extremity and with (b) the nBR and (c) intraoral histological findings. We hypothesized a priori that there would be a difference between DPN patients and healthy participants regarding the electrophysiological and functional somatosensory and structural assessment of the trigeminal region.

## Results

### Sample characteristics

**Figure 1:**
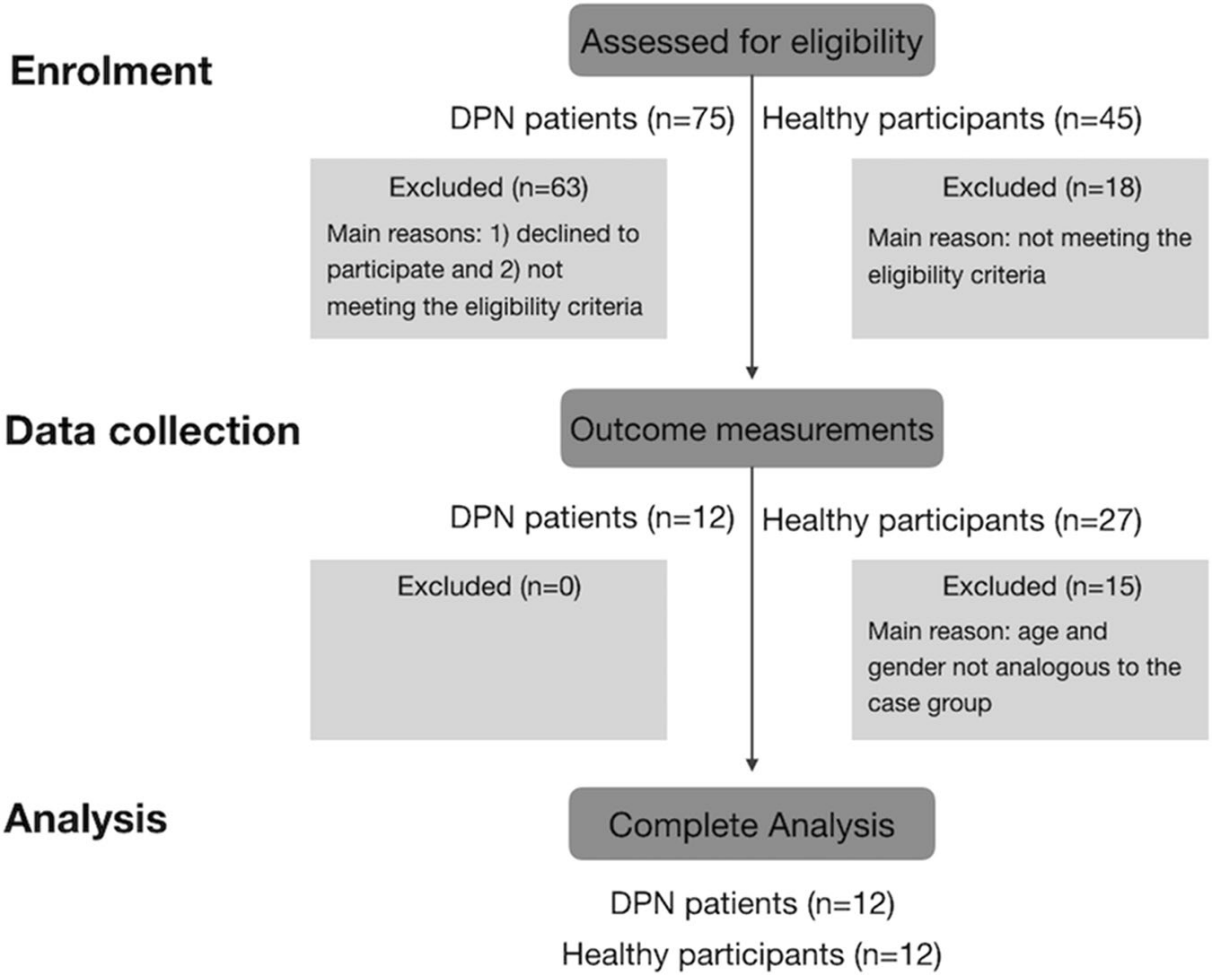
Patients and healthy participants flow throughout the study.

Seventy-five patients and 45 healthy participants were invited to participate, but only 12 were included for complete analysis in each group (Fig. 1). The mean age and standard deviation (SD) of the DPN patients (8 women, 4 men) and healthy participants (7 women, 5 men) were 63.0 (7.0) and 59.5 (9.1) (p = 0.307), respectively. All patients had type 1 diabetes and a confirmed diagnosis of DPN. Furthermore, half of the patients had painful DPN while the other half had non-painful DPN (see Table 1 for a clinical description of pain-related information for each patient).

**Table 1:** Clinical description of pain-related information for each enrolled patient.

Patient ID number	Pain Intensity (NRS)^a^	Pain Duration (months)	BPI-DPN Intensity^b^	BPI-DPN Interference	DN4^c^
#1	0	0	0	0	0
#2	0	0	0	0	2
#3	6	48	8	6.14	6
#4	0	0	0	0	1
#5	0	0	2	0.14	0
#6	5	30	5	0.14	5
#7	0	0	0	0	1
#8	0	0	0	0	1
#9	5	132	0	0	8
#10	6	24	8	6.14	4
#11	7	48	8	6.71	4
#12	4	6	6	5.33	4

BPI-DPN = Brief Pain Inventory for Painful Diabetic Peripheral Neuropathy, DN4 = *Douleur Neuropathique en 4 Questions*.

^a^NRS = 0–10 numeric rating scale. The patients were asked to score the average pain in the last 24 hours.

^b^See ref.^37^ for more information.

^c^See ref.^36^ for more information.

### nBR assessment

**Figure 2:**
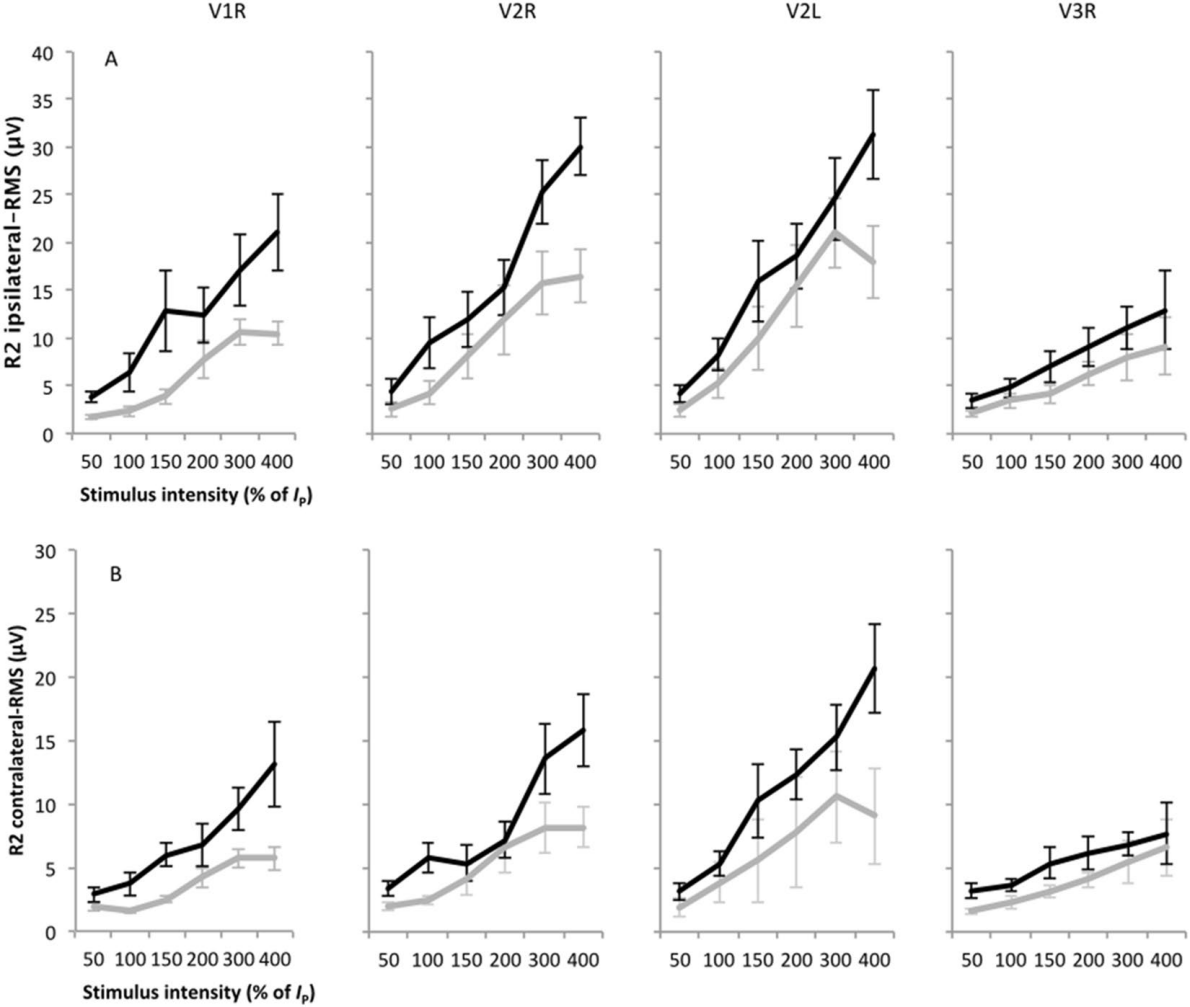
Electromyography (EMG) records of the ipsilateral (**A**) and contralateral (**B**) R2 response, quantified as the root mean square (RMS) at different intensities of stimulation, from 50 to 400% of pain threshold (*I*_P_) for the diabetic peripheral neuropathy (DPN) patients and healthy participants considering the three branches of the trigeminal nerve, i.e., right supraorbital (V1R), infraorbital (V2R) and the mental (V3R) nerve and also the left infraorbital (V2L) nerve. DPN patients showed overall higher EMG amplitude (ANOVA: F = 26.77, p < 0.001) but without significant interactions among group vs. site vs. intensity (p < 0.050). A similar pattern was also found for the area-under-the-curve (AUC) values.

A detailed assessment of the nBR is presented in Table 2 (analysis of variance (ANOVA) - absolute values) and Table 3 (latency). DPN patients presented higher sensory (ANOVA: F = 5.65, p = 0.027) and pain thresholds to electrical stimulation (ANOVA: F = 5.53, p = 0.029) when compared to healthy participants. Likewise, DPN patients showed overall higher electromyography (EMG) amplitude (ANOVA: F = 26.8, p < 0.001) and area-under-the-curve (AUC) (ANOVA: F = 28.3, p < 0.001) when compared with healthy participants, although without significant interactions among group, intensity and site (p > 0.050 - Fig. 2). However, there were no differences between the groups regarding the pain intensity scores and latency (p > 0.050) (Tables 2–3). Finally, analysis of covariance (ANCOVA) did not show any main effect or interaction of the covariate pain threshold (F < 3.74, p > 0.069).

**Table 2:** ANOVA results comparing groups, site of stimulation, side of recording and intensity of stimulation for different nociceptive blink reflex (nBR) parameters.

	*I*_0_ (mA)	*I*_P_ (mA)	Pain (VAS)	RMS (µV)	AUC(µV x ms)	Latency (ms)
**Main effects**						
**Factors**						
1-Group	**F** = **5**.**04**,**p** = **0**.036*	**F** = **5**.**42**,**p** = **0**.**031**	F = 0.00,p = 0.976	**F** = **21**.**52**,**p** < **0**.**001**	**F** = **22**.**09**,**p** < **0**.**001**	F = 1.50,p = 0.235
2-Site	F = 1.11,p = 0.348	**F** = **2**.**39**,**p** = **0**.**040**	**F** = **3**.**38**,**p** = **0**.**027**	**F** = **14**.**78**,**p** < **0**.**001**	**F** = **16**.**62**, **p** < **0**.**001**	**F** = **10**.**90**, **p** < **0**.**001**
3-Side	NA	NA	NA	**F** = **83**.**04**,**p** < **0**.**001**	**F** = **86**.**08**,**p** < **0**.**001**	**F** = **225**.**70**,**p** < **0**.**001**
4-Intensity	NA	NA	**F** = **104**.**47**,**p** < **0**.**001**	**F** = **101**.**05**,**p** < **0**.**001**	**F** = **101**.**78**,**p** < **0**.**001**	**F** = **10**.**90**,**p** = **0**.**004**
**Interactions**						
1 × 2	**F** = **3**.**14**,**p** = **0**.**031**	F = 1.87, p = 0.152	**F** = **4**.**54**,**p** = **0**.**007**	F = 0.37,p = 0.772	F = 0.47,p = 0.698	**F** = **3**.**40**,**p** = **0**.**025**
1 × 3	NA	NA	NA	F = 2.17,p = 0.163	F = 1.72,p = 0.211	F = 1.60,p = 0.223
1 × 4	NA	NA	F = 0.28,p = 0.886	F = 2.31,p = 0.053	**F** = **2**.**37**,**p** = **0**.**048**	F = 0.00,p = 0.976
2 × 3	NA	NA	NA	**F** = **4**.**23**,**p** = **0**.**010**	**F** = **4**.**44**,**p** = **0**.**008**	F = 0.10,p = 0.943
2 × 4	NA	NA	F = 1.59,p = 0.097	**F** = **2**.**79**,**p** = **0**.**001**	**F** = **3**.**32**,**p** < **0**.**001**	F = 0.70,p = 0.530
3 × 4	NA	NA	NA	**F** = **8**.**90**, **p** < **0**.**001**	**F** = **7**.**07**,**p** < **0**.**001**	F = 0.30,p = 0.597
1 × 2 × 3	NA	NA	NA	F = 0.76,p = 0.521	F = 0.86,p = 0.470	F = 0.80,p = 0.520
1 × 2 × 4	NA	NA	F = 0.95,p = 0.492	F = 1.25,p = 0.232	F = 1.09,p = 0.361	F = 0.60,p = 0.640
1 × 3 × 4	NA	NA	NA	F = 1.72,p = 0.141	F = 1.08,p = 0.375	F = 0.00,p = 0.954
2 × 3 × 4	NA	NA	NA	F = 0.70,p = 0.775	F = 0.75,p = 0.728	F = 0.90,p = 0.469
1 × 2 × 3 × 4	NA	NA	NA	F = 0.84,p = 0.622	F = 0.73,p = 0.746	F = 0.60,p = 0.609

^*^Bold cells present significant p-values (p < 0.050).

*I*_0_ = electrical sensory threshold, *I*_P_ = electrical pinprick threshold, VAS = visual analogue scale, RMS = root mean square, AUC = area-under-the-curve, NA = Not applicable.

**Table 3:** Mean and standard error of mean (SEM) of nociceptive blink reflex (nBR) latency.

	200% of *I*_P_		300% of *I*_P_	
	Ipsilateral	Contralateral	Ipsilateral	Contralateral
**V1R**				
DPN patients	43.7 (1.0)	44.9 (0.9)	43.2 (1.4)	44.4 (1.3)
Controls	42.7 (0.5)	43.8 (0.4)	41.8 (0.7)	43.3 (0.5)
**V2R**				
DPN patients	42.5 (0.8)	43.2 (0.9)	41.5 (0.9)	42.6 (1.0)
Controls	41.9 (0.5)	43.2 (0.4)	40.7 (0.5)	42.3 (0.6)
**V2L**				
DPN patients	43.6 (0.9)	44.7 (0.9)	42.7 (0.7)	43.8 (0.8)
Controls	42.6 (0.7)	43.7 (0.7)	41.3 (0.6)	42.5 (0.7)
**V3R**				
DPN patients	46.8 (0.7)	48.1 (0.9)	45.4 (0.9)	46.2 (0.9)
Controls	42.9 (0.7)	44.2 (0.7)	43.0 (0.5)	44.1 (0.5)

*I*_P_ = pain threshold, V1R = right supraorbital nerve, V2R = right infraorbital nerve, V2L = left infraorbital nerve, V3R = right mandibular nerve. DPN = diabetic peripheral neuropathy.

### Somatosensory functional and structural assessment

The absolute QST data and Z-scores for both groups and sites are described in Table 4. As expected, all patients had altered somatosensory sensitivity in the lower limb, most commonly in tactile sensitivity (MDT), but also including thermal thresholds (CDT and WDT) (Table 2). Patients were less sensitive to cold and warm (CDT - p = 0.045 and WDT - p = 0.007) and to mechanical non-painful and painful stimuli (MDT - p < 0.001, VDT - p < 0.001 and MPT - p = 0.042) and reported higher occurrence of PHS (p = 0.019) when compared with healthy participants (Table 4), indicating an overall low somatosensory sensitivity on the distal leg. On the other hand, the intraoral somatosensory sensitivity alterations were less pronounced and were presented in the thermal assessment (Table 4). Patients had lower WDT (p = 0.004) and reported higher occurrence of PHS (p = 0.040) when compared to healthy participants (Table 4). Furthermore, there was a tendency towards statistical significantly lower intraoral tactile sensitivity in DPN patients when compared to healthy participants (p = 0.053).

**Table 4:** Mean values and standard deviations (SD) of the quantitative sensory testing (QST) absolute data and Z-scores from the buccal mucosa of the posterior mandibular region and lateral dorsum of the foot in patients with diabetic peripheral neuropathy (DPN) and healthy participants with analogous age and sex (controls).

QST - intraoral	DPN patients (n = 12)	Controls (n = 12)	p-value^a^	Cohen’s d^a^
**Absolute values**/**Z-Scores**				
CDT (°C)	22.8 (11.1)/−0.2 (1.3)	22.7 (9.1)/−0.2 (1.3)	0.951	—
WDT (°C)	45.2 (2.5)/1.0 (1.4)	47.7 (1.3)/−0.3 (0.7)	0.004^b^	1.26
TSL (°C)	25.9 (10.7)/0.0 (1.4)	28.0 (9.8)/−0.3 (1.0)	0.478	—
PHS (x/3)^c^	0.6 (0.7)	0.08 (0.2)	0.040^c^	—
CPT (°C)	9.7 (8.5)/0.7 (1.4)	4.6 (6.6)/−0.0 (1.1)	0.235	—
HPT (°C)	48.4 (1.2)/−0.2 (0.99)	48.8 (1.4)/0.1 (1.1)	0.433	—
MDT (mN)	38.5 (53.2)/−1.3 (1.3)	7.1 (10.3)/−0.3 (1.1)	0.053	—
MPT (mN)	139.8 (236.6)/0.1 (1.5)	136.0 (171.8)/−0.3 (1.0)	0.321	—
MPS (VAS)	1.8 (2.0)/−0.2 (1.1)	2.4 (3.0)/0.0 (1.0)	0.628	—
ALL (VAS)	0.05 (0.09)	0.01 (0.02)	0.149	—
WUR (VAS)	2.5 (1.4)/−0.3 (2.0)	2.5 (1.0)/0.0 (0.7)	0.557	—
VDT (x/8)	5.9 (1.1)/−0.8 (1.5)	6.5 (0.7)/−0.0 (0.9)	0.116	—
PPT (kPa)	142.8 (53.8)/−0.4 (1.3)	136.0 (49.9)/−0.2 (1.1)	0.794	—
**QST – distal leg**				
CDT (°C)	20.8 (9.1)/−1.4 (1.1)	29.2 (1.2)/0.2 (0.5)	0.045	0.86
WDT (°C)	45.4 (2.7)/−1.59 (0.57)	41.6 (3.3)/−0.9 (0.7)	0.007	−1.56
TSL (°C)	21.6 (11.8)/−1.1 (0.7)	12.1 (3.2)/−0.3 (0.4)	0.004	−1.36
PHS (x/3)^c^	1.2 (1.1)	0.2 (0.3)	0.019	—
CPT (°C)	5.03 (9.3)/−0.5 (1.0)	3.9 (6.6)/−0.6 (0.6)	0.737	—
HPT (°C)	48.2 (2.1)/−0.4 (1.3)	47.8 (1.3)/−0.8 (0.7)	0.859	—
MDT (mN)	217.5 (304.1)/−3.1 (1.4)	5.9 (3.4)/−0.5 (0.7)	<0.001	−2.75
MPT (mN)	589.6 (228.0)/−2.6 (0.72)	386.6 (234)/−1.8 (0.8)	0.042	−0.90
WUR (VAS)	7.6 (17.2)/0.8 (1.4)	5.2 (7.9)/0.5 (1.4)	0.808	—
VDT (x/8)	4.1 (1.3)/−2.2 (1.3)	7.5 (0.9)/0.5 (0.9)	<0.001	2.80
PPT (kPa)	494.5 (161.2)/0.3 (1.8)	636.8 (175.3)/−0.4 (0.88)	0.069	—

^a^p-values and effect sizes (Cohen’s d – calculated only for significant mean differences) were computed based on the log_10_ transformed values, with the exception of CPT, HPT and VDT.

^b^Significant differences (p < 0.050).

^c^PHS differences were compared using Man-Whitney U test (p < 0.050).

**Figure 3:**
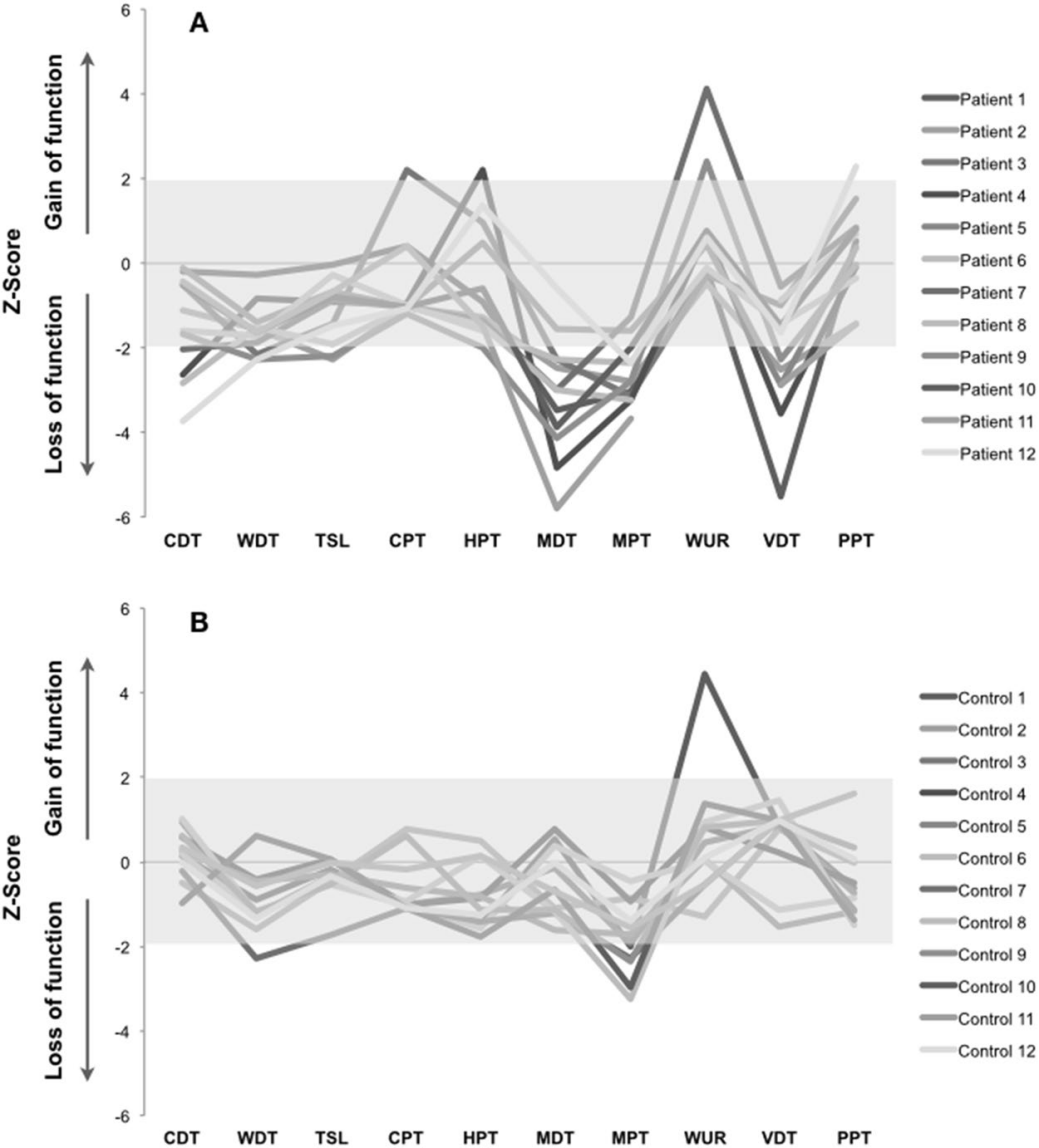
Somatosensory profiles from the distal leg of diabetic peripheral neuropathy patients (**A**) and healthy participants with analogous age and sex (**B**). The gray zone indicates a Z-score between −1.96 and 1.96, representing the normal range level of the reference group. A Z-score above 1.96 indicates a gain in somatosensory function and a Z-score below −1.96 indicates loss of somatosensory function. CDT = cold detection threshold; WDT = warm detection threshold; TSL = thermal sensory limen; CPT = cold pain threshold; HPT = heat pain threshold; MDT = mechanical detection threshold; MPT = mechanical pain threshold; MPS = mechanical pain sensitivity; WUR = wind-up ratio; VDT = vibration detection threshold; PPT = pressure pain threshold.

**Figure 4:**
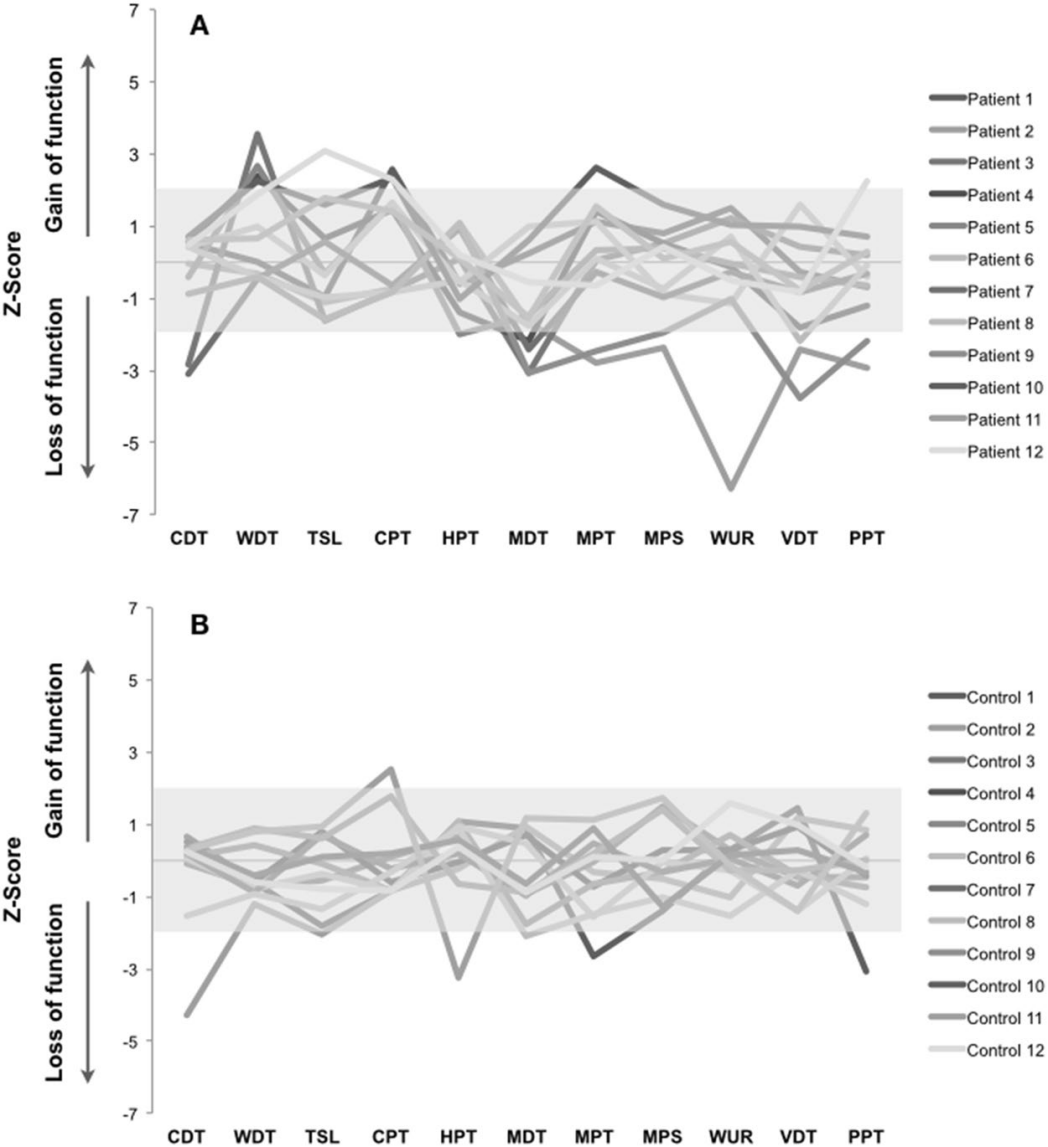
Somatosensory profiles from the buccal mucosa of diabetic peripheral neuropathy patients (**A**) and healthy participants with analogous age and sex (**B**). The gray zone indicates a Z-score between −1.96 and 1.96, representing the normal range level of the reference group. A Z-score above 1.96 indicates a gain in somatosensory function and a score below −1.96 indicates loss of somatosensory function. CDT = cold detection threshold; WDT = warm detection threshold; TSL = thermal sensory limen; CPT = cold pain threshold; HPT = heat pain threshold; MDT = mechanical detection threshold; MPT = mechanical pain threshold; MPS = mechanical pain sensitivity; WUR = wind-up ratio; VDT = vibration detection threshold; PPT = pressure pain threshold.

Individual somatosensory profiles for both groups and sites and frequencies of absolute abnormalities of intraoral QST (Z-scores outside 95% confidence interval (CI) of reference data) are shown, respectively, in Figures 3 and 4 and Table 5. Loss of mechanical somatosensory function (91% or 11 out of 12 patients) was the most common somatosensory abnormality presented in the lower limb of DPN patients (Fig. 3). The most frequent intraoral somatosensory absolute abnormalities found in the DPN group were thermal hyperalgesia (41.7% or 5 out of 12 patients) and mechanical hypoesthesia (33.3% or 4 out of 12 patients) (Fig. 4 and Table 5). As expected and due to natural variation and the tissue characteristics, some abnormalities (values outside 95% CI) were also found in the control group^16,21^ (Table 5). However, the frequency of no intraoral somatosensory abnormalities at all (L0G0) was significantly lower in the DPN patients when compared to the reference group (p = 0.013) (Table 5). In addition, the cumulative frequency of DPN patients presenting somatosensory loss without any gain (L1G0, L2G0 or L3G0) presented a tendency towards statistical significantly higher proportion when compared to the reference group, respectively, 42% and 11% (p = 0.079) (Table 5).

**Table 5:** Frequency of absolute somatosensory abnormalities according to the LossGain scores in patients with diabetic peripheral neuropathy (DPN) and in the reference healthy group.

Loss	Gain				
	G0 (no)	G1 (thermal)	G2 (mechanical)	G3 (both)	All
**DPN patients (n** = **12)**					
L0 (no)	**1 (8**.**3%)**	1 (8.3%)	4 (33.4%)	0 (0.0%)	6 (50%)
L1 (thermal)	*0 (0.0%)*	0 (0.0%)	0 (0.0%)	0 (0.0%)	0 (0.0%)
L2 (mechanical)	*4 (33.4%)*	0 (0.0%)	0 (0.0%)	0 (0.0%)	4 (33.4%)
L3 (both)	*1 (8.3%)*	0 (0.0%)	1 (8.3%)	0 (0.0%)	2 (16.6%)
All	6 (50.0%)	1 (8.3%)	5 (41.7%)	0 (0.0%)	12 (100%)
**Reference (n** = **27)**					
L0 (no)	**14 (52%)**	2 (7.4%)	8 (29.6%)	0 (0.0%)	24 (92.5%)
L1 (thermal)	*1 (3.7%)*	0 (0.0%)	0 (0.0%)	0 (0.0%)	1 (3.7%)
L2 (mechanical)	*2 (7.4%)*	0 (0.0%)	0 (0.0%)	0 (0.0%)	2 (7.4%)
L3 (both)	*0 (0.0%)*	0 (0.0%)	0 (0.0%)	0 (0.0%)	0 (0.0%)
All	18 (66.6%)	2 (7.4%)	9 (29.6%)	0 (0.0%)	27 (100%)

Bold cells indicate significant differences between groups for that specific LossGain coding category, i.e., L0G0 (p = 0.013). Italic highlights the cumulative frequency of somatosensory loss without any gain, which presented a tendency towards statistical significance between groups (p = 0.079).

Mean (SD) fibre length density of the foot epidermis (IENFD) and buccal mucosa (NFLD) were lower in DPN patients when compared to healthy participants, respectively, 88.0 (64.6) vs. 614 (218) mm^2^, (p < 0.001 – distal leg) and 501 (279) vs. 696 (164) mm^2^ (p = 0.048 – buccal mucosa).

Finally, the correlation between the intraoral QST and nBR recordings at 200% of *I*_P_ and intraoral NFLD outcomes can be found as Supplementary Tables S1–S2. Likewise, the correlation between QST Z-scores of buccal mucosa and distal leg can be found as Supplementary Tables S3 and S4. These exploratory analyses showed that there were only few significant correlations (5%) (p < 0.050 – Supplementary Tables S1–S4).

## Discussion

The primary aim of the present case-control study was to compare the trigeminal nociceptive function, intraoral somatosensory profile and structural nerve changes between DPN patients and healthy participants. The main findings were: (a) DPN patients presented trigeminal hyperexcitability, i.e., higher EMG amplitude and AUC for the nBR; (b) DPN patients showed decreased NFLD, and (c) the intraoral somatosensory profile was not substantially changed in DPN patients. However, loss of intraoral somatosensory function occurred more often in DPN patients compared to healthy participants.

Blink reflex using standard electrodes and the supraorbital nerve as a stimulation site has long been used to evaluate patients with diabetes in order to detect subclinical signs of cranial neuropathy^22^. Most of the studies have reported prolonged latencies and decreased amplitude of the R2 component in diabetes patients, regardless of the presence of DPN^20,23,24^. However, there are no published studies where the trigeminal function was assessed with the nBR evoked by electrical stimuli in DPN patients. Even though nBR and BR are similar tests, the afferent arch of the reflex might not be analogous. Validation and methodological studies of nBR have shown that the mean latencies are above 40 ms whereas the mean latencies of BR lie around 33 ms^8,9,25^. Such differences could argue in favour of the preferable activation of nociceptive A-delta afferents and pathways of subnucleus caudalis of the spinal trigeminal nucleus for the nBR, whereas A-beta fibres activation and the principal sensory trigeminal nucleus are the preferable for the BR. We did not find significantly prolonged R2 latencies in the DPN group compared with healthy, although, there is a possibility that the sample size was too small to be able to detect significant differences (type II error). In addition, a previous study that investigated the corneal reflex in 21 DPN patients also did not find prolonged latencies when compared to healthy participants^26^. The corneal reflex could be considered analogous to the nBR, except that purely nociceptive responses are involved with the former^27^, whereas contamination of non-nociceptive afferents are often unavoidable with the nBR evoked by electrical stimulation^28^.

On the other hand, significant differences between groups were found for the amplitude (RMS and AUC) of the R2 component of the nBR. However, the higher amplitudes of the EMG responses are indicative of neuronal facilitation, which contradicts previous evidence where the amplitude of the BR is reduced in DPN patients^20^, though most of the studies have focused only on the latency analysis^18,23,24,29^. The significantly higher pain thresholds might also be a possible explanation for the higher EMG amplitude, though covariance analysis did not suggest significant effects of the pain threshold. Probably, the different interneurons composing the sensorial arch of the nBR and BR can account for these differences, which may also explain the abovementioned lack of significantly prolonged R2 responses when nociceptive afferents are primarily recruited. Furthermore, neuronal depression, e.g., loss of sensory function and neuronal hyperexcitability, e.g., pain processing amplification are indeed part of the complex scenario of DPN symptomatology^30–33^.

Although sensorial symptoms of oral hypoesthesia and hyperalgesia have been reported for patients with DPN or long-term diabetes^34–36^, this is the first study that performed the full QST battery in order to assess the intraoral sensory profile of DPN patients. Previous evidence has shown hypoalgesia to pinprick pain in patients with DPN^37^. On the other hand, our results showed higher intraoral sensitivity for the detection of warm sensation and a tendency for mechanical hypoesthesia in DPN patients. Likewise, individual Z-scores and the LG coding indicated an occurrence of loss of intraoral somatosensory function in the DPN patients that should not be ignored (42%), with only one DPN patient presenting intraoral QST values within the normative range. Loss of somatosensory function has also been reported in asymptomatic type 1 diabetic children^38^, which reinforces the value of intraoral QST for the early detection of signs of neuropathic alterations. In addition, considering that the somatosensory profiling presented no consistent picture, further investigations with a more robust sample will be needed to confirm these findings. Finally, the stimulation of oral mucosa with pinpricks may have caused sensitization in healthy participants that reported allodynia during MPS, which resulted in more than expected (26%) of just mechanical somatosensory gain (G2) in the healthy group. Since the histological characteristics of the oral mucosa are different from the skin, methodological concerns should be taken into consideration when performing and judging intraoral QST.

Another novelty of this study is the structural assessment of the intraoral nerve fibres. Skin biopsy procedures, in order to quantify small nerve fibres, can be used as an objective measurement of nerve density and as an accurate indication of loss of neuronal tissue^17^. The literature reports a severe nerve fibre loss at the distal leg, as measured by IENFD^22^. Here we show, for the first time, that DPN patients, in addition to loss of IENFD at the leg, also have a significant reduction in NFLD in the oral mucosa, albeit these abnormalities are not as profound as they are at the distal leg. These findings support the evidence of the distal sensory nerves of the lower extremities as the most affected in diabetic patients^1,19,38^.

The lack of relevant linear correlations between intraoral somatosensory findings and the nBR and NFLD indicate that these outcomes might partially represent independent features within the complex scenario of neuropathy. This lack of association has also been reported in previous studies, where somatosensory function and fibre density was not correlated in the assessment of the distal leg in patients with DPN^39–41^, although the opposite is also reported, i.e., a significant correlation between somatosensory function and nerve fibre density^42,43^. The explanation for lack of relationship between nerve function and histological features is currently not known, but one possibility is that increased activity in subpopulations of regenerating fibres may mask a loss of somatosensory function^44^. Furthermore, it has been suggested that the nerve fibre density has little or nothing to do with the function of the remaining nerve fibres, which can be everything from hypo- to hyperactive, or even completely normal^45^. Lastly, a recent systematic review concluded that the chances of finding a positive association between the structure and function of nerve fibres is much higher when the two tests (i.e. skin biopsy and QST) are performed at the exact same anatomical site^46^.

This study has some limitations that should be addressed. (A) The sample size could be considered small to detect systematic somatosensory differences between DPN patients and healthy participants, which are expected to be smaller differences when considering an apparently “unaffected” region. However, the sample size was large enough to indicate trigeminal nociceptive processing differences. In addition, this study was part of a bigger project with type 1 DPN patients and the oral assessment was performed after 4–5 hours of experiments in the leg region. This particular situation hampered the recruitment and compliance of patients. (B) The lack of patient-oriented outcomes, e.g., oral health related quality of life, and a comprehensive oral assessment, even though symptoms of intraoral pain were not reported. (C) Only type 1 diabetic patients were assessed, so the generalization to other patient groups should be made with caution. Nevertheless, we believe that the present study has demonstrated important and novel findings that will need to be tested in larger-scale studies.

The orofacial somatosensory and neurophysiological consequences of DPN appear to present heterogeneous characteristics, considering that not only signs of enhanced trigeminal nociceptive function, but also loss of intraoral nerve fibre length density can be identified along with minor somatosensory alterations.

## Materials and Methods

### Sample and Ethics

The source populations for this case-control study were all the adult patients diagnosed with type 1 diabetes that were registered in the database of Aarhus University Hospital (cases) and the general adult population of Aarhus municipality (controls). This study took place at the Department of Dentistry and Oral Health, Aarhus University and the participants were recruited from January 2015 until April 2016 through random invitation letters (cases) and advertisements (controls).

This study was performed in accordance with the Helsinki Declaration II and had the approval from the Regional Ethics Committee as well as the Danish Data Protection Agency. All participants gave their voluntary consent after a full explanation of all procedures.

### Eligibility criteria

Inclusion criteria for the case group (n = 12) were: a confirmed clinical DPN diagnosis with or without pain^47–49^ and a Michigan Neuropathy Screening Instrument (MNSI) score ≥4^50^. In addition, the exclusion criteria for the case group were: endocrine disorders, other than DM, or neurological disorders, other than neuropathy (e.g. Parkinson, multiple sclerosis, dementia), amputation or foot ulcers, diagnosed psychological or personality disorders, pace-maker, pregnancy and inability to follow or understand the research procedures, in particular the QST instructions. A comprehensive clinical examination was used to assess the eligibility criteria of the case group. In all patients, nerve conduction tests in sural sensory, tibial and peroneal motor and median and ulnar sensory and motor nerves were performed. DPN diagnosis was confirmed in case of at least two abnormal nerves of which one should be the sural nerve.

Healthy participants (control group) with similar age- and sex-distribution to the case group (n = 12) were recruited based on the following criteria: absence of serious dental or medical illness, e.g., orofacial pain or chronic headaches, regular intake of medication, such as antidepressants, anticonvulsants or non-steroidal anti-inflammatories and psychiatric or personality disorders. A detailed interview/anamnesis was used to assess the eligibility criteria of the control group.

### Variables

The primary and secondary outcomes were measured in the following order: (a) distal leg QST (secondary; b) NFLD of the distal leg (secondary); (c) intraoral QST (primary); (b) nBR (primary) and (c) NFLD of the buccal mucosa (secondary). All the procedures were performed in a single session (approximately 5 h) for all healthy controls and for 42% of the DPN patients. For the remaining 58% of patients, a second session, not more than 10 weeks later, was arranged to take the oral mucosa biopsy.

### QST

The somatosensory assessment was made on the left distal leg within the lateral malleolus and the left buccal mucosa, in accordance with the standardized German Research Network on Neuropathic Pain (DFNS), which also presented acceptable values of reliability for intraoral evaluation^10,13^. A detailed description of the full QST battery can be found elsewhere^12,13^. In brief, 13 parameters, which assemble a comprehensive evaluation of the somatosensory submodalities, i.e., sensitivity to touch, vibration, temperature and pain, were measured in the following order: cold detection threshold (CDT), WDT, thermal sensory limen (TSL) and the number PHS during the procedure, followed by cold pain threshold (CPT) and heat pain threshold (HPT) which were measured with the aid of a computerized thermal stimulator, PATHWAY (MEDOC, Ramat Yishai, Israel)^11–13^.

MDT was determined using a standardized set of von Frey filaments (OptiHair2, MARSTOCKnervtest, Marburg, Germany), which apply forces between 0.25 mN and 512 mN. The modified “method of limits” technique, which applies an “up-down rule”, was used to determine the threshold^11–13^. The mechanical pain threshold (MPT) was measured using a standardized set of 7 custom-made weighted pinprick stimulators (manufactured at Aarhus University, Aarhus, Denmark) with fixed stimulus intensities (8, 16, 32, 64, 128, 256 and 512 mN) and a flat contact surface (diameter of 0.2 mm). Also, the same modified “method of limits” technique was used to determine the threshold^11–13^.

Suprathreshold measurements, i.e., mechanical pain sensitivity (MPS) and dynamic mechanical allodynia (DMA) were determined only intraorally using the same weighted pinprick and three light tactile stimulators: a cotton wisp, a cotton wool tip and a toothbrush^11–13^. Each of the seven pinprick stimuli and the three tactile stimuli were applied five times in a balanced order and the subjects were asked to give a pain rating for each stimulus on a 0–100 numerical rating scale (from 0 = ‘no pain’ to 100 = ‘most pain imaginable’). The geometric mean of all pain ratings for pinprick and light touch were considered to determine, respectively, the MPS and DMA^11–13^. In the sequence, a single pinprick stimulus and 10 pinprick stimuli with the same force, repeated at a rate of 1 Hz, were applied to determine the wind-up ratio (WUR). The mean pain rating of three series of the train stimulus divided by the mean pain rating of three single stimuli (train/single pinprick) was considered the WUR^11–13^.

A Rydel–Seiffer tuning fork (64 Hz, 8/8 scale) was set in motion and left in place until the participant could not feel vibration anymore. Thus, the vibration detection threshold (VDT) was calculated as the mean disappearance threshold of three stimulus repetitions^11–13^. Finally, the pressure pain threshold (PPT) was measured with a digital pressure algometer (SOMEDIC Algometer ®, SOMEDIC Sales AB, Sweden). The participants were instructed to press a button at the first painful sensation. The PPT was determined as the arithmetic mean of three repetitions^11–13^.

### nBR

A detailed description of the nBR assessment, which presented acceptable values of reliability, can be found elsewhere^8^. In brief, the nBR was recorded by placing two surface self-adhesive EMG electrodes (Neuroline 720, Ambu ®, Denmark) on both orbicularis oculi muscles. The recorded signals were amplified and band-pass filtered between 20–1000 Hz and the sampling rate was 2000 Hz (Nicolet Viking™, Natus Medical Inc., USA). A custom built planar concentric electrode was used to elicit the nBR by stimulation of all three branches of the trigeminal nerve^9^.

Each stimulus sweep consisted of a train of three pulses with duration of 0.3 ms and an inter-pulse interval of 3 ms and was applied to the skin directly above the entry zones of the right supraorbital (V1R), infraorbital (V2R) and the mental (V3R) nerves and also the left infraorbital (V2L) nerve in a randomized order^6–8^.

The individual *I*_0_ and *I*_P_ to the electrical stimulation were determined for each site before the nBR recordings by the application of an up-down staircase method consisting of 5 series of ascending and descending stimuli (0.2 mA increment rate)^6–8,51^.

For each site, the nBR recordings comprised a total of 6 stimulation blocks with 6 individual sweeps each at an interstimulus interval (ISI) of approximately 15–17 s^6–8^. The intensities of the blocks were also applied in a randomized order considering the following: 50, 100, 150, 200, 300 and 400% of *I*_P_. To avoid contamination with the startle reaction and the related R3 responses, the first stimulus of each block was announced to the participant. Furthermore, the participants were asked to score the stimulus-evoked pain intensity at the end of each block with the aid of a 0–10 numerical rating scale (NRS) with 0 indicating no pain at all and 10 indicating worst pain imaginable. Thus, the following variables were obtained: EMG records of the R2 component of the nBR, quantified as the root mean square (RMS) (µV) and area-under-the-curve (AUC) (µV × ms) of the rectified and averaged sweeps in the time window from 27–87 ms; onset latencies (ms) of the R2 responses at 200 and 300% of *I*_P_ measured for the averaged sweeps and the stimulus-evoked pain intensity (NRS)^6–8^.

### Skin biopsies (IENFD and NFLD)

Two biopsies were obtained from each study participant: one from the right distal leg (8–10 cm above the lateral malleolus, the exact same anatomical site where QST was performed) and another one from the left buccal mucosa adjacent to the 2^nd^ mandibular molar. The biopsies were taken under sterile conditions and subcutaneous anaesthesia (leg biopsy) or a buccal nerve block with lidocaine 4% (intraoral biopsy) with a 3-mm disposable biopsy punch (Miltex, York, PA). The biopsies were fixated in Zamboni’s fixative overnight and cryoprotected in 20% glycerol and 0.08 M Sorenson’s PO_4_ buffer overnight. From each biopsy, three 50-μm-cryostat sections (Microm Cryostat M 500 OM, Zeiss, Germany) were immunostained with rabbit anti-human PGP 9.5 (1:1000; Zytomed Systems, Berlin, Germany) as a primary antibody and horseradish peroxidase-marked goat anti-rabbit as a secondary antibody (1:200; Vector Laboratories, Burlingame, CA). IENFD was assessed from the leg biopsy only, but because of the natural structure of the mucosal tissue, it was not possible to estimate IENFD as often done with skin biopsies. NFLD was, however, assessed in both biopsies. The microscopical analysis was performed using an Olympus BX51 microscope (60 × oil immersion lens (Olympus UPlanSApo; NA = 1.35) and newCAST stereological software (Visiopharm, Hoersholm, Denmark) in a blinded fashion. A detailed methodological description of both the IENFD and NFLD measurements can be found elsewhere, but here we include a brief description of the NFLD estimation and the settings used^17,52^.

**Figure 5:**
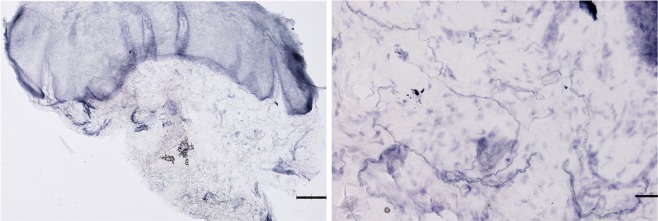
Left: overview (x4 objective lens) of a 50 μm thick section from buccal mucosa biopsy from a representative healthy participant. Scale bar: 30 μm. Middle: visible nerve fibres from the same section (x20 objective lens) in the lower mucosa. Scale bar: 200 μm. Right: example of a severe loss of nerve fibres in the lower mucosa in a DPN patient (x20 objective lens). The sections were stained using PGP 9.5 antibody.

To obtain isotropy of the test planes and to estimate the natural tubular shape of the nerve fibres in the thick sections, a virtual plane probe was used. The region of interest was defined as the area from the mucosa-submucosa junction and as deep down as possible towards the submucosa, or up to 200 micrometres (Fig. 5)^17^. Briefly, 3D sampling boxes were superimposed over the tissue section, thereby generating randomized isotropic virtual planes in systematically sampled fields of view^17^. The sampling box height was set to 15 μm and the box area size to 4.800 μm^2^. Sampling steps were 85 × 70 μm (*in the x and y-direction)*, and a plane separation distance of 25 μm.

### Statistics

Quantitative variables (age, QST, nBR and NFLD) were reported as means and SD or standard error of the mean (SEM) and the sex was reported in numeric values and percentage. All the quantitative variables were assessed for normal distribution using the Kolmogorov-Smirnov test and, when applicable, a log_10_ transformation was performed when the results were significant, considering an alpha level of 5% (p < 0.050). Thus, the following variables were log10 transformed: absolute QST values, i.e., raw data, of the CDT, WDT, MDT, MPT, MPS, WUR and PPT; absolute nBR values, i.e., raw data of *I*_0_, *I*_P_, NRS, RMS, AUC and latency.

QST data were transformed into Z-values according to the following expression: Z-score = (Participant_value_ − Mean_reference_)/SD _reference_^12^. In cases where QST parameters were not normally distributed, log-transformed values were used to compute the Z-values. A Z-score of 0 ± 1.96 represents the interval which includes 95% of the healthy reference data. Positive Z-scores indicate a gain of somatosensory function for the tested stimuli, whereas negative Z-scores indicate a loss of somatosensory function and a Z-score of 0 corresponds to the mean value of the reference data^12^. Reference data for the distal leg QST were automatically generated from the QST managing software eQUISTA **®** (StatConsult, Magdeburg, Germany). On the other hand, reference data for the intraoral QST were computed from 27 healthy participants of the dataset of the Section of Orofacial Pain and Jaw Function, Department of Dentistry and Oral Health, Aarhus University (Fig. 1). In addition, intraoral QST Z-scores were grouped according to presence of absolute somatosensory abnormalities (LossGain coding) if the individual Z-values were outside of the 95% confidence interval of the reference group, which yields the identification of no loss of sensitivity (L0), loss of thermal sensitivity only (L1), loss of mechanical sensitivity only (L2), mixed loss of sensitivity (L3), no gain of sensitivity (G0), gain of thermal sensitivity only (G1), gain of mechanical sensitivity only (G2), and gain of sensitivity to both thermal and mechanical stimuli (G3)^21^.

A t-test for independent samples was computed to compare QST (absolute values) and NFLD between groups, except the PHS, which was computed using the Mann-Whitney U test. In addition, Fisher’s exact test was computed to compare the distribution of somatosensory abnormalities categories according to the LossGain system^21^. The significance level was set at 5% (p = 0.050).

ANOVA with the following factors, group (2 levels), site (4 levels - V1, V2R, V2L and V3), side (2 levels - ipsilateral and contralateral) and intensity (6 levels - 50 to 400%), was performed to compare the RMS and AUC of nBR. Additionally, ANCOVA with the *I*_P_ as covariate was performed to evaluate the possible influence of the pain threshold on the RMS and AUC values between the groups. Finally, ANOVA with the following factors, group (2 levels), site (4 levels - V1, V2R, V2L and V3), side (2 levels - ipsilateral and contralateral) and intensity (2 levels - 200 and 300%), was performed to compare the latency of the nBR. When appropriate, post hoc analyses were performed using Tukey’s Honestly Statistical Difference (HSD). The significance level was set at 5% (p = 0.050).

The Pearson product-moment correlation coefficient was computed to associate the intraoral QST absolute values with the nBR recordings at 200% of *I*_P_ and intraoral NFLD outcomes. In addition, we also testes for correlation of the QST Z-scores between test sites, i.e., distal leg and buccal mucosa. Due to the explorative nature of these secondary analyses, no adjustment for multiple comparisons was made for a total of 480 correlations, 240 for each group. Thus, the significance level was also set at 5% (p = 0.050).

## Supplementary information

Supplementary Tables

## Data Availability

The datasets generated during and/or analysed during the current study are available from the corresponding author upon reasonable request.
