# Supplementary material for: Trigeminal nociceptive function and oral somatosensory functional and structural assessment in patients with diabetic peripheral neuropathy
Source: Sci Rep. 2019 Jan 17;9:169. doi: 10.1038/s41598-018-37041-4 (PMC6336810; doi:10.1038/s41598-018-37041-4)
Supplement: Supplementary file 1 — Supplementary Tables [file 41598_2018_37041_MOESM1_ESM.docx]

**Trigeminal nociceptive function and oral somatosensory functional and structural assessment in patients with diabetic peripheral neuropathy**

**Authors:** YM Costa, P Karlsson, LR Bonjardim, PCR Conti, H Tankisi, TS Jensen, JR Nyengaard, P Svensson, L Baad-Hansen.

Table S1. Pearson product-moment correlation coefficients between intraoral quantitative sensory testing (QST) and the nociceptive blink reflex (nBR) responses at 200% of electrical pinprick threshold (*I*_P_) and nerve fibre length density (NFLD) of the buccal mucosa of diabetic peripheral neuropathy patients.

|  | *I*_0_ | *I*_P_ | RMS (Ipsi.) | RMS (Contra.) | AUC (Ipsi.) | AUC (Contra.) | Latency (Ipsi.) | Latency (Contra.) | NFLD |
| --- | --- | --- | --- | --- | --- | --- | --- | --- | --- |
| r-value  p-value | | | | | | | | | |
| CDT | -0.1834 | -0.2948 | -0.4966 | -0.1821 | -0.4791 | -0.2147 | -0.1668 | -0.1860 | 0.1582 |
|  | p=0.568 | p=0.352 | p=0.101 | p=0.571 | p=0.115 | p=0.503 | p=0.604 | p=0.563 | p=0.623 |
| WDT | -0.1443 | -0.0021 | 0.1296 | 0.0718 | 0.1664 | 0.0609 | -0.4607 | -0.4209 | -0.1527 |
|  | p=0.654 | p=0.995 | p=0.688 | p=0.824 | p=0.605 | p=0.851 | p=0.132 | p=0.173 | p=0.636 |
| TSL | 0.2418 | 0.0883 | 0.3053 | 0.4062 | 0.3297 | 0.4161 | 0.1520 | 0.2682 | 0.4586 |
|  | p=0.449 | p=0.785 | p=0.335 | p=0.190 | p=0.295 | p=0.178 | p=0.637 | p=0.399 | p=0.134 |
| CPT | -0.4062 | -0.3904 | -0.3432 | -0.2082 | -0.3895 | -0.2378 | 0.1814 | 0.1441 | 0.1390 |
|  | p=0.190 | p=0.210 | p=0.275 | p=0.516 | p=0.211 | p=0.457 | p=0.573 | p=0.655 | p=0.667 |
| HPT | 0.1048 | 0.2466 | 0.2227 | -0.0362 | 0.2563 | -0.0525 | -0.3300 | -0.3610 | -0.3464 |
|  | p=0.746 | p=0.440 | p=0.487 | p=0.911 | p=0.421 | p=0.871 | p=0.295 | p=0.249 | p=0.270 |
| MDT | 0.3900 | 0.3313 | 0.2406 | 0.0630 | 0.2837 | 0.0987 | -0.2050 | -0.2170 | -0.1788 |
|  | p=0.210 | p=0.293 | p=0.451 | p=0.846 | p=0.372 | p=0.760 | p=0.523 | p=0.498 | p=0.578 |
| MPT | -0.0366 | 0.0372 | 0.1739 | 0.0087 | 0.2280 | 0.0035 | -0.4189 | -0.5007 | -0.0704 |
|  | p=0.910 | p=0.909 | p=0.589 | p=0.979 | p=0.476 | p=0.991 | p=0.175 | p=0.097 | p=0.828 |
| MPS | -0.0036 | -0.1069 | -0.4474 | -0.3436 | -0.5030 | -0.3343 | 0.5644 | 0.4977 | -0.0706 |
|  | p=0.991 | p=0.741 | p=0.145 | p=0.274 | p=0.096 | p=0.288 | p=0.056 | p=0.100 | p=0.827 |
| ALL | 0.2470 | 0.2326 | -0.1943 | -0.0400 | -0.2159 | -0.0539 | 0.2741 | 0.2809 | -0.2505 |
|  | p=0.439 | p=0.467 | p=0.545 | p=0.902 | p=0.500 | p=0.868 | p=0.389 | p=0.377 | p=0.432 |
| WUR | 0.2155 | 0.2071 | -0.3384 | -0.2923 | -0.3809 | -0.2654 | 0.3006 | 0.3105 | -0.0814 |
|  | p=0.501 | p=0.518 | p=0.282 | p=0.357 | p=0.222 | p=0.405 | p=0.342 | p=0.326 | p=0.801 |
| VDT | -0.1724 | -0.1217 | 0.0989 | 0.1481 | 0.0278 | 0.1255 | 0.4742 | 0.5551 | 0.0706 |
|  | p=0.592 | p=0.706 | p=0.760 | p=0.646 | p=0.932 | p=0.697 | p=0.119 | p=0.061 | p=0.827 |
| PPT | 0.1297 | -0.0056 | 0.4702 | 0.5421 | 0.5121 | 0.5509 | -0.0676 | 0.0396 | 0.5216 |
|  | p=0.688 | p=0.986 | p=0.123 | p=0.069 | p=0.089 | p=0.063 | p=0.835 | p=0.903 | p=0.082 |

CDT = cold detection threshold; WDT = warm detection threshold; TSL = thermal sensory limen; CPT = cold pain threshold; HPT = heat pain threshold; MDT = mechanical detection threshold; MPT = mechanical pain threshold; MPS = mechanical pain sensitivity; ALL = dynamic mechanical allodynia; WUR = wind-up ratio; VDT = vibration detection threshold; PPT = pressure pain threshold. *I*_0_ = electrical sensory threshold; RMS = root mean square; AUC = area-under-the-curve. Ipsi. = ipsilateral side of stimulation; Contra. = contralateral side of stimulation.

Table S2. Pearson product-moment correlation coefficients between intraoral quantitative sensory testing (QST) and the nociceptive blink reflex (nBR) responses at 200% of electrical pinprick threshold (*I*_P_) and nerve fibre length density (NFLD) of the buccal mucosa of healthy participants.

|  | *I*_0_ | *I*_P_ | RMS (Ipsi.) | RMS (Contra.) | AUC (Ipsi.) | AUC (Contra.) | Latency (Ipsi.) | Latency (Contra.) | NFLD |
| --- | --- | --- | --- | --- | --- | --- | --- | --- | --- |
| r-value  p-value | | | | | | | | | |
| CDT | -0.1274 | 0.1147 | 0.2349 | 0.2880 | 0.2647 | 0.2861 | **-0.7037 *** | **-0.6594** | 0.1804 |
|  | p=0.709 | p=0.737 | p=0.487 | p=0.390 | p=0.432 | p=0.394 | **p=0.016** | **p=0.027** | p=0.596 |
| WDT | -0.0058 | -0.0045 | -0.3310 | -0.4135 | -0.3229 | -0.4086 | 0.4095 | 0.3632 | 0.2626 |
|  | p=0.987 | p=0.990 | p=0.320 | p=0.206 | p=0.333 | p=0.212 | p=0.211 | p=0.272 | p=0.435 |
| TSL | 0.0513 | 0.0772 | -0.1084 | -0.1844 | -0.1195 | -0.1721 | 0.3944 | 0.4210 | 0.0128 |
|  | p=0.881 | p=0.822 | p=0.751 | p=0.587 | p=0.726 | p=0.613 | p=0.230 | p=0.197 | p=0.970 |
| CPT | 0.2772 | 0.1825 | 0.4823 | 0.5425 | 0.4311 | 0.5208 | -0.1385 | -0.2489 | **-0.6696** |
|  | p=0.409 | p=0.591 | p=0.133 | p=0.085 | p=0.186 | p=0.100 | p=0.685 | p=0.460 | **p=0.024** |
| HPT | -0.3016 | -0.1918 | -0.2670 | -0.1964 | -0.2351 | -0.1562 | -0.2903 | -0.1428 | 0.3008 |
|  | p=0.367 | p=0.572 | p=0.427 | p=0.563 | p=0.486 | p=0.647 | p=0.387 | p=0.675 | p=0.369 |
| MDT | 0.3141 | -0.0334 | -0.1587 | -0.0905 | -0.1739 | -0.0868 | 0.0173 | -0.0267 | -0.4168 |
|  | p=0.347 | p=0.922 | p=0.641 | p=0.791 | p=0.609 | p=0.800 | p=0.960 | p=0.938 | p=0.202 |
| MPT | -0.1531 | 0.1577 | 0.1198 | 0.1210 | 0.1368 | 0.1493 | -0.2507 | -0.2068 | 0.3806 |
|  | p=0.653 | p=0.643 | p=0.726 | p=0.723 | p=0.688 | p=0.661 | p=0.457 | p=0.542 | p=0.248 |
| MPS | -0.2486 | -0.3534 | -0.2823 | -0.2242 | -0.3161 | -0.2408 | -0.2389 | -0.5393 | -0.1971 |
|  | p=0.461 | p=0.286 | p=0.400 | p=0.507 | p=0.344 | p=0.476 | p=0.479 | p=0.087 | p=0.561 |
| ALL | -0.2072 | -0.2065 | 0.2133 | 0.3342 | 0.2123 | 0.3385 | -0.1799 | -0.2124 | -0.2740 |
|  | p=0.541 | p=0.542 | p=0.529 | p=0.315 | p=0.531 | p=0.308 | p=0.597 | p=0.531 | p=0.415 |
| WUR | -0.2401 | -0.1286 | -0.2181 | -0.2592 | -0.1772 | -0.2736 | -0.3285 | -0.2879 | 0.3689 |
|  | p=0.477 | p=0.706 | p=0.519 | p=0.442 | p=0.602 | p=0.416 | p=0.324 | p=0.391 | p=0.264 |
| VDT | 0.4513 | 0.5705 | 0.3123 | 0.1239 | 0.3019 | 0.1001 | 0.0628 | 0.1867 | 0.1658 |
|  | p=0.164 | p=0.067 | p=0.350 | p=0.717 | p=0.367 | p=0.770 | p=0.854 | p=0.583 | p=0.626 |
| PPT | 0.1485 | 0.4201 | 0.3553 | 0.3296 | 0.3512 | 0.3442 | -0.5523 | -0.3985 | 0.2037 |
|  | p=0.663 | p=0.198 | p=0.284 | p=0.322 | p=0.290 | p=0.300 | p=0.078 | p=0.225 | p=0.548 |

* Bold cells present significant p-values (p<0.050).

CDT = cold detection threshold; WDT = warm detection threshold; TSL = thermal sensory limen; CPT = cold pain threshold; HPT = heat pain threshold; MDT = mechanical detection threshold; MPT = mechanical pain threshold; MPS = mechanical pain sensitivity; ALL = dynamic mechanical allodynia; WUR = wind-up ratio; VDT = vibration detection threshold; PPT = pressure pain threshold. *I*_0_ = electrical sensory threshold; RMS = root mean square; AUC = area-under-the-curve. Ipsi. = ipsilateral side of stimulation; Contra. = contralateral side of stimulation.

Table S3. Pearson product-moment correlation coefficients between intraoral and distal leg quantitative sensory testing (QST) of diabetic peripheral neuropathy patients.

| Leg    Oral | CDT | WDT | TSL | CPT | HPT | PHS | MDT | MPT | WUR | VDT | PPT |
| --- | --- | --- | --- | --- | --- | --- | --- | --- | --- | --- | --- |
|  |  |  |  |  | **r-value**  **p-value** |  |  |  |  |  |  |
| CDT | -0.1956 | -0.5791 | -0.0081 | -0.4107 | 0.1385 | -0.1784 | 0.2588 | 0.4058 | -0.4862 | -0.4154 | 0.5143 |
|  | p=0.588 | p=0.079 | p=0.982 | p=0.238 | p=0.703 | p=0.622 | p=0.470 | p=0.245 | p=0.154 | p=0.233 | p=0.128 |
| WDT | -0.2605 | 0.2007 | 0.3808 | -0.4138 | **0.9076 *** | **0.6413** | -0.0039 | -0.1364 | 0.0060 | -0.0093 | 0.3300 |
|  | p=0.467 | p=0.578 | p=0.278 | p=0.235 | **p=0.000** | **p=0.046** | p=0.991 | p=0.707 | p=0.987 | p=0.980 | p=0.352 |
| TSL | 0.1324 | -0.0282 | 0.2236 | 0.4560 | 0.1502 | -0.0976 | -0.3645 | 0.2005 | -0.3353 | -0.2592 | -0.0049 |
|  | p=0.715 | p=0.938 | p=0.535 | p=0.185 | p=0.679 | p=0.789 | p=0.300 | p=0.579 | p=0.344 | p=0.470 | p=0.989 |
| CPT | 0.2860 | **-0.7300** | **-0.6763** | -0.0496 | -0.1975 | -0.4647 | 0.1721 | -0.0086 | 0.0642 | -0.5094 | 0.0400 |
|  | p=0.423 | **p=0.017** | **p=0.032** | p=0.892 | p=0.584 | p=0.176 | p=0.634 | p=0.981 | p=0.860 | p=0.133 | p=0.913 |
| HPT | 0.1028 | 0.4985 | 0.4012 | 0.0512 | 0.4860 | **0.7122** | 0.0523 | -0.0247 | 0.2892 | 0.2572 | 0.2214 |
|  | p=0.777 | p=0.143 | p=0.251 | p=0.888 | p=0.154 | **p=0.021** | p=0.886 | p=0.946 | p=0.418 | p=0.473 | p=0.539 |
| MDT | -0.2700 | 0.5226 | 0.5191 | 0.1565 | -0.2390 | 0.1157 | -0.0267 | 0.0253 | 0.1758 | 0.2154 | -0.2143 |
|  | p=0.451 | p=0.121 | p=0.124 | p=0.666 | p=0.506 | p=0.750 | p=0.942 | p=0.945 | p=0.627 | p=0.550 | p=0.552 |
| MPT | 0.0602 | 0.4780 | 0.6263 | -0.1010 | 0.4010 | 0.5330 | -0.2522 | -0.0850 | 0.1043 | 0.0298 | 0.4180 |
|  | p=0.869 | p=0.162 | p=0.053 | p=0.781 | p=0.251 | p=0.113 | p=0.482 | p=0.815 | p=0.774 | p=0.935 | p=0.229 |
| MPS | 0.1265 | -0.4661 | -0.5492 | 0.3741 | -0.6050 | **-0.7754** | 0.2266 | 0.2171 | -0.0525 | -0.1512 | -0.5397 |
|  | p=0.728 | p=0.175 | p=0.100 | p=0.287 | p=0.064 | **p=0.008** | p=0.529 | p=0.547 | p=0.886 | p=0.677 | p=0.107 |
| ALL | 0.3874 | -0.2917 | -0.3415 | 0.2173 | -0.0894 | -0.3597 | 0.4339 | 0.3792 | 0.0147 | -0.0344 | -0.1298 |
|  | p=0.269 | p=0.413 | p=0.334 | p=0.547 | p=0.806 | p=0.307 | p=0.210 | p=0.280 | p=0.968 | p=0.925 | p=0.721 |
| WUR | -0.0809 | -0.3099 | -0.5769 | 0.3935 | -0.3353 | **-0.7088** | 0.2494 | 0.1340 | -0.0710 | -0.0007 | **-0.8396** |
|  | p=0.824 | p=0.384 | p=0.081 | p=0.261 | p=0.344 | **p=0.022** | p=0.487 | p=0.712 | p=0.846 | p=0.999 | **p=0.002** |
| VDT | 0.4249 | -0.2863 | **-0.7253** | 0.3305 | -0.2860 | -0.3325 | -0.1820 | -0.2096 | 0.0110 | 0.0949 | -0.3385 |
|  | p=0.221 | p=0.423 | **p=0.018** | p=0.351 | p=0.423 | p=0.348 | p=0.615 | p=0.561 | p=0.976 | p=0.794 | p=0.339 |
| PPT | 0.0254 | 0.0492 | 0.5038 | -0.2936 | 0.2781 | 0.4901 | -0.0341 | 0.0294 | 0.1573 | -0.3874 | **0.7520** |
|  | p=0.944 | p=0.893 | p=0.138 | p=0.410 | p=0.437 | p=0.150 | p=0.926 | p=0.936 | p=0.664 | p=0.269 | **p=0.012** |

* Bold cells present significant p-values (p<0.050).

CDT = cold detection threshold; WDT = warm detection threshold; TSL = thermal sensory limen; CPT = cold pain threshold; HPT = heat pain threshold; MDT = mechanical detection threshold; MPT = mechanical pain threshold; MPS = mechanical pain sensitivity; ALL = dynamic mechanical allodynia; WUR = wind-up ratio; VDT = vibration detection threshold; PPT = pressure pain threshold.

Table S4. Pearson product-moment correlation coefficients between intraoral and distal leg quantitative sensory testing (QST) of healthy participants.

| Leg    Oral | CDT | WDT | TSL | CPT | HPT | PHS | MDT | MPT | WUR | VDT | PPT |
| --- | --- | --- | --- | --- | --- | --- | --- | --- | --- | --- | --- |
|  |  |  |  |  | **r-value**  **p-value** |  |  |  |  |  |  |
| CDT | **0.6230 *** | **-0.6018** | -0.4456 | **-0.6518** | 0.4408 | **-0.6253** | -0.2038 | 0.2226 | 0.3954 | 0.0312 | 0.1120 |
|  | **p=0.030** | **p=0.038** | p=0.147 | **p=0.022** | p=0.151 | **p=0.030** | p=0.525 | p=0.487 | p=0.203 | p=0.923 | p=0.729 |
| WDT | -0.3502 | **0.5783** | 0.4573 | -0.2805 | 0.2599 | 0.4170 | -0.3845 | 0.1048 | 0.0373 | 0.1186 | 0.5742 |
|  | p=0.264 | **p=0.049** | p=0.135 | p=0.377 | p=0.415 | p=0.177 | p=0.217 | p=0.746 | p=0.908 | p=0.714 | p=0.051 |
| TSL | -0.4245 | 0.4770 | 0.2945 | 0.1424 | 0.0965 | 0.3961 | -0.2343 | 0.1741 | 0.3194 | 0.0201 | 0.2688 |
|  | p=0.169 | p=0.117 | p=0.353 | p=0.659 | p=0.765 | p=0.202 | p=0.464 | p=0.588 | p=0.312 | p=0.950 | p=0.398 |
| CPT | 0.3870 | -0.4036 | -0.4367 | 0.3310 | -0.5750 | -0.3248 | 0.4008 | 0.0064 | -0.1880 | 0.3955 | **-0.7000** |
|  | p=0.214 | p=0.193 | p=0.156 | p=0.293 | p=0.050 | p=0.303 | p=0.197 | p=0.984 | p=0.558 | p=0.203 | **p=0.011** |
| HPT | -0.1772 | 0.2473 | 0.4035 | -0.4444 | **0.7897** | 0.0797 | -0.1791 | 0.4105 | 0.4398 | -0.3155 | **0.8780** |
|  | p=0.582 | p=0.438 | p=0.193 | p=0.148 | **p=0.002** | p=0.806 | p=0.578 | p=0.185 | p=0.153 | p=0.318 | **p=0.000** |
| MDT | 0.1508 | 0.0910 | 0.3393 | -0.2506 | 0.1876 | -0.4347 | 0.5270 | 0.4115 | -0.0226 | -0.0952 | 0.3427 |
|  | p=0.640 | p=0.779 | p=0.281 | p=0.432 | p=0.559 | p=0.158 | p=0.078 | p=0.184 | p=0.944 | p=0.768 | p=0.275 |
| MPT | 0.0643 | 0.3179 | 0.2955 | -0.2276 | 0.4484 | 0.2416 | -0.4763 | 0.2386 | **0.6418** | -0.2827 | **0.5889** |
|  | p=0.843 | p=0.314 | p=0.351 | p=0.477 | p=0.144 | p=0.449 | p=0.118 | p=0.455 | **p=0.024** | p=0.373 | **p=0.044** |
| MPS | **0.5891** | -0.1032 | -0.2406 | 0.2673 | -0.5226 | -0.1219 | 0.1600 | -0.3007 | -0.4517 | 0.1035 | -0.5563 |
|  | **p=0.044** | p=0.749 | p=0.451 | p=0.401 | p=0.081 | p=0.706 | p=0.619 | p=0.342 | p=0.140 | p=0.749 | p=0.060 |
| ALL | 0.1837 | -0.1324 | 0.0696 | 0.3909 | 0.1864 | 0.0099 | 0.4346 | -0.1090 | -0.0282 | -0.4352 | -0.1308 |
|  | p=0.568 | p=0.682 | p=0.830 | p=0.209 | p=0.562 | p=0.976 | p=0.158 | p=0.736 | p=0.931 | p=0.157 | p=0.685 |
| WUR | 0.1818 | -0.3902 | -0.3865 | -0.4103 | 0.3598 | -0.3869 | -0.3014 | -0.1956 | 0.0673 | -0.1166 | -0.0602 |
|  | p=0.572 | p=0.210 | p=0.215 | p=0.185 | p=0.251 | p=0.214 | p=0.341 | p=0.542 | p=0.835 | p=0.718 | p=0.853 |
| VDT | -0.3712 | 0.0261 | -0.1919 | 0.0976 | 0.1522 | -0.0512 | -0.4590 | 0.1272 | 0.4230 | 0.2466 | 0.1008 |
|  | p=0.235 | p=0.936 | p=0.550 | p=0.763 | p=0.637 | p=0.874 | p=0.133 | p=0.694 | p=0.171 | p=0.440 | p=0.755 |
| PPT | 0.1364 | -0.0850 | -0.0619 | -0.4314 | 0.5505 | -0.3034 | -0.3919 | **0.6464** | **0.9331** | -0.0066 | **0.5921** |
|  | p=0.673 | p=0.793 | p=0.848 | p=0.161 | p=0.064 | p=0.338 | p=0.208 | **p=0.023** | **p=0.000** | p=0.984 | **p=0.043** |

* Bold cells present significant p-values (p<0.050).

CDT = cold detection threshold; WDT = warm detection threshold; TSL = thermal sensory limen; CPT = cold pain threshold; HPT = heat pain threshold; MDT = mechanical detection threshold; MPT = mechanical pain threshold; MPS = mechanical pain sensitivity; ALL = dynamic mechanical allodynia; WUR = wind-up ratio; VDT = vibration detection threshold; PPT = pressure pain threshold.
